# Altitude Cure for a Dog of High Degree

**Published:** 1914-04

**Authors:** 


Altitude Cure for a Dog of High Degree. H. Grenier de Gardenal, *Jour. de Med. de Bordeaux,* August 24, 1913, describes the luxurious quarters of a dog, who, not thriving in the fogs of London, was taken to the beneficial climate of the Pyrenees by "his father and mother, I should say his masters." Allusion is made to race suicide, etc.

